# 

**Published:** 1890-01-04

**Authors:** 


					S'SUM
S1THOMAfS Host PI T.AL
Tun Norwich hospi tal
Glasgow Western Infirmary
NEW YEAR'S SPECIAL NUMBER
[One Penny.
Saturday, January 4, 1890.

				

